# Vibrotactile display design: Quantifying the importance of age and various factors on reaction times

**DOI:** 10.1371/journal.pone.0219737

**Published:** 2019-08-09

**Authors:** Tian Bao, Lydia Su, Catherine Kinnaird, Mohammed Kabeto, Peter B. Shull, Kathleen H. Sienko

**Affiliations:** 1 Dept. of Mechanical Engineering, University of Michigan, Ann Arbor, Michigan, United States of America; 2 Internal Medicine, University of Michigan, Ann Arbor, Michigan, United States of America; 3 State Key Laboratory of Mechanical System and Vibration, Shanghai Jiao Tong University, Shanghai, China; Université catholique de Louvain, BELGIUM

## Abstract

Numerous factors affect reaction times to vibrotactile cues. Therefore, it is important to consider the relative magnitudes of these time delays when designing vibrotactile displays for real-time applications. The objectives of this study were to quantify reaction times to typical vibrotactile stimuli parameters through direct comparison within a single experimental setting, and to determine the relative importance of these factors on reaction times. Young (n = 10, 21.9 ± 1.3 yrs) and older adults (n = 13, 69.4 ± 5.0 yrs) performed simple reaction time tasks by responding to vibrotactile stimuli using a thumb trigger while frequency, location, auditory cues, number of tactors in the same location, and tactor type were varied. Participants also performed a secondary task in a subset of the trials. The factors investigated in this study affected reaction times by 20–300 ms (reaction time findings are noted in parentheses) depending on the specific stimuli condition. In general, auditory cues generated by the tactors (<20 ms), vibration frequency (<20 ms), number of tactors in the same location (<30 ms) and tactor type (<50 ms) had relatively small effects on reaction times, while stimulus location (20–120 ms) and secondary cognitive task (>130 ms) had relatively large effects. Factors affected young and older adults’ reaction times in a similar manner, but with different magnitudes. These findings can inform the development of vibrotactile displays by enabling designers to directly compare the relative effects of key factors on reaction times.

## Introduction

Vibrotactile displays, one common type of haptic displays [1], have been applied to various areas of the body to deliver spatial and temporal information in a variety of real-time applications including sensory augmentation devices [2,3]. During driving, vibrotactile displays provide navigation information [4] and warn drivers of potential collisions [5,6]. During flying, they can provide altitude information, warning signals and simple communication to replace or reinforce visual and auditory cues [7,8]. Vibrotactile displays have also been applied to virtual reality applications to enhance physical sensations within a virtual environment [9,10]. Additionally, vibrotactile displays have been studied as a method for conveying eyes-free information for multiple applications including for people with visual impairments [11–13]. More recently, vibrotactile displays have been used for training arm movements, balance, and gait [14–22].

Depending on the application, human reaction times (RTs) to vibrotactile stimuli have been shown to vary from 200 ms to 2000 ms [4,5,22–25]. Delays caused by RTs to vibrotactile cues should be considered when designing vibrotactile displays because the time required to perceive, process, and subsequently act in response to a vibrotactile cue will affect the utility of the cue based on the specific application. For example, surgical applications often require small, carefully controlled movements, therefore a fast RT (less than 250 ms) is desirable [23]. Likewise, fast RTs are also desirable for golf swing training (e.g., initiation of downswing through impact typically takes less than 250 ms [24]) and step response (e.g., step response to unpredictable, abrupt backward translation of a support surface typically takes less than 300 ms [22]) applications. On the other hand, relatively longer RTs are acceptable for use in driving and flying applications as warning signals (e.g., ~900 ms [5,25] and ~1600 ms [4], respectively). Lastly, RTs associated with real-time gait training applications vary considerably based on the specific activity; step/stride frequencies for walking and running usually range from 1 Hz to 3 Hz requiring RTs ranging from 300 to 1000 ms.

Numerous individual factors have been shown to affect RTs. Harrar and Harris investigated RTs to vibrotactile stimuli across various body locations (forehead, lip, neck, hand, and foot) and showed that RTs increased proportionally with the distance from the brain with a slope of 45 ms/m [26]. Shull et al. found that RTs for arm movements were ~60 ms faster than leg movements by placing tactors (i.e., vibrotactile transducers) on the arm and leg, respectively [27]. Asseman et al. investigated the effects of location and stimulus-response compatibility on RTs while participants were exposed to an unpredictable translation of the support surface. Their results indicated that 1) RTs to vibrotactile stimuli displayed on the head were ~60 ms faster than those displayed on the sternum during a protective stepping task and 2) RTs to vibrotactile stimuli displayed on the forehead were ~100 ms faster than those displayed on the back of the head for the same task [22]. Peon and Prattichizzo demonstrated that a higher vibrotactile stimuli intensity produced faster (i.e., ~25 ms) RTs compared with a low stimuli intensity [23]. Hick showed that RTs logarithmically increased as the number of different visual stimuli increased in choice experiments [28]. Haggerty et al. showed that verbal and push-button reaction times increased by up to 200 ms when participants were asked to simultaneously respond to torso-based vibrotactile cues to make postural corrections [29]. Although limited studies have investigated the effects of aging on RTs to vibrotactile stimuli, Taware et al. demonstrated significant increases in RTs (i.e., on the order of 60 ms) to both auditory and visual stimuli for older adults (50–59 yrs) compared to young adults (20–29 yrs) [30]. Ng and Chan reported that multiple factors including age, gender, and level of education had significant effects on RTs for auditory and visual stimuli; in general, aging increased RTs, while females had faster RTs than males, and participants that had completed either a tertiary or secondary education level had faster RTs than participants that had only completed a primary education level [31]. Additionally, they studied RTs to vibrotactile stimuli applied at the wrist and leg locations; however, no significant differences were observed based on stimuli location [31].

Additional factors including frequency of stimulation and tactor type have been shown to affect human responses to vibrotactile stimuli. Vibrotactile displays typically use one of two types of tactors: linear actuators or rotary motors [2,32]. In a study comparing two types of linear actuators, Lee et al. showed that the C-2 tactor induced larger non-volitional postural shifts in the directions of the applied stimuli (on the torso) than the Tactaid tactor [33].

The majority of RT studies have focused on visual and auditory stimuli [34]. Among the studies that have investigated RTs to vibrotactile stimuli, the most commonly performed studies have compared RTs to vibrotactile stimuli with RTs to visual and/or auditory stimuli [4,26,31,34,35], or have compared RTs to vibrotactile stimuli while varying one-to-two stimuli factors (e.g., location, secondary task) using the same subject population and/or experimental condition [5,22,23,26,27,29,34]. Therefore, there are limited quantitative data that facilitate the determination of the relative importance of vibrotactile stimuli factors on RTs. As previously mentioned, RTs are important to consider when designing vibrotactile displays for various applications; specific applications may necessitate careful selection of tactor locations, number of tactors, and type of tactors. To inform vibrotactile display design for real-time applications, this study quantitatively examined RTs to vibrotactile stimuli as a function of various factors through direct comparison within a single experimental setting in young adults and older adults using simple reaction time tasks. The factors investigated in this study included stimulus frequency, type of tactor that generates the vibrotactile stimulus, stimulus location, auditory cues generated by the vibration (ACV), number of tactors in the same location, and secondary task. Furthermore, the effect of varying the number of possible stimuli locations on RTs was studied.

## Material and method

This study was performed in four parts; the first three experimental parts were performed within a single session on one day and the fourth part was performed during a separate session on a subsequent day. Throughout the experiments, vibrotactile stimuli were presented using different tactor types at different locations on the body and tactors were secured by Velcro straps. A shim was used to achieve approximately uniform pressure across all body location sites. Participants were asked to confirm that the pressure of the tactor against their skin felt similar at all locations.

During the experiments, participants were barefoot. They were instructed to stand with their arms held at their sides with their feet hip-width apart at a 15° lateral rotation angle [33] while looking straight ahead at a stationary, eye-level visual target. Participants were asked to respond as quickly as possible each time they perceived a vibrotactile stimulus by pressing a thumb trigger with their dominant hand. Trigger responses were recorded by a computer and RT was defined as the time when the command to the tactor was applied to the time the trigger signal was received by the computer. Participants were not informed of their performance during the experiments. Before the start of the experiments, participants were asked to familiarize themselves with the stimuli-trigger system by practicing responses prior to each experimental part for approximately 3–4 minutes. All participants were asked to confirm they could feel the vibration at all locations at approximately the same intensity prior to each experimental part.

Ten healthy young adults (YA) (21.9 ± 1.3 yrs; six males, four females) and ten healthy community-dwelling older adults (OA) (68.3 ± 2.7 yrs; four males, six females) participated in the first three parts of this study. All participants completed the three parts in the same order. Nine healthy OA (70.5 ± 5.6 yrs; four males, five females), including six participants who also participated in the first three parts of the study, participated in the fourth part of this study. Individuals were eligible to participate in this study if they reported no neurological conditions or joint replacements, and if they could stand for at least 1 min without assistance. Prior to participation, all participants completed the 10-g monofilament test [36] on the dorsal aspect of the dominant foot and were excluded if they failed the test. Both YA and OA followed the same experimental protocol. All participants gave written informed consent and the study was conducted in accordance with the Declaration of Helsinki. The study was reviewed and approved by the University of Michigan Institutional Review Board (HUM00020302).

### Part I: Effects of frequency and tactor type

The first part of the study was designed to test the effects of vibration frequency and tactor types on RTs. Four types of vibrotactile stimuli were presented using three different tactors as shown in Table 1. The C-2 tactor (EAI, Inc.) operated at either 250 Hz or 200 Hz with a 6.2 cm^2^ contact area and 0.5 cm^2^ contactor area. Only the contactor vibrated to provide vibrotactile stimuli. The Tactaid VBW32 tactor (Audiological Engineering Corp) operated at 250 Hz with a 3.7 cm^2^ contact area. Based on experimental measurements, the coin-style motors used in this study (Precision Microdrives, 310–101 vibration motor) operated at a frequency of 215 ± 5 Hz with an input of 3.7 V. They were encapsulated in a plastic case with a contact area of 6.2 cm^2^, and have previously been used in a smartphone balance trainer device [37]. Both the C-2 and Tactaid tactors were driven by a sinusoidal signal generated at an RMS current of 0.225 A (or peak-to-peak voltage of 4.47 V) by the tactor controller unit provided by EAI, Inc. The amplitude of the signals, measured as peak-to-peak displacement of the C2 and Tactaid tactors, was approximately 200 μm and 50 μm, respectively [33]. The coin-style motors were driven by a battery at 3.7 V with vibration amplitude of 0.8 G. The battery was fully charged prior to each data collection session.

**Table 1:** Major differences among the three tactors.

Type	Transducer display	Frequency (Hz)	Area (cm^2^)	Motor Response Time
C-2 tactor	Linear actuator	250/200*	0.5 (6.2**)	< 33 ms
Tactaid tactor	Linear actuator	250	3.7	—***
Coin-style motor	Rotary motor	200	6.2	< 91 ms

* C-2 tactor vibrated at 250 or 200 Hz depending on the trial

** The contactor area was 0.5 cm^2^, while the contact area was 6.2 cm^2^

*** The response time was not found from the specification sheet

**Fig 1:**
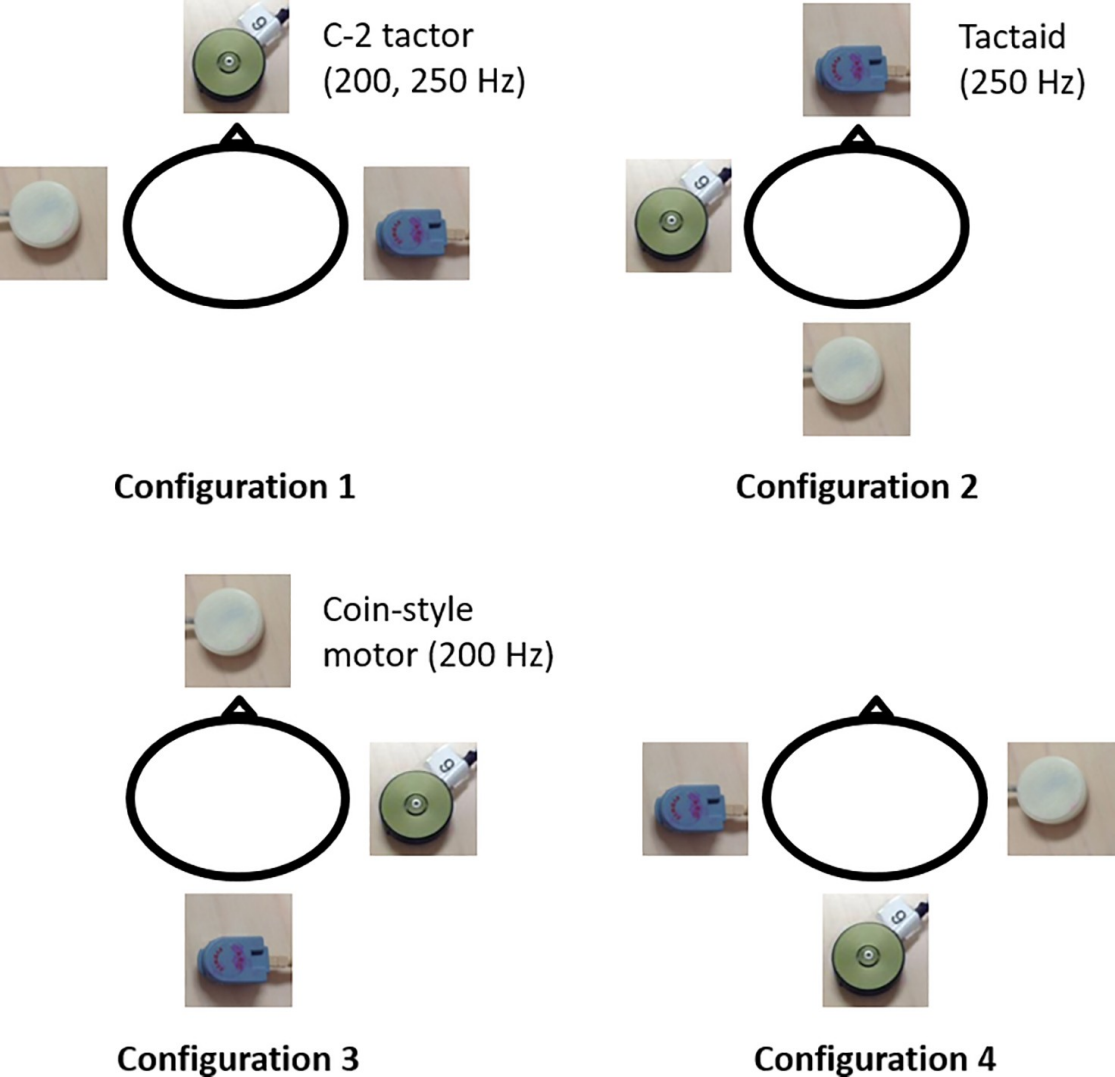
Experimental illustration for Part I: Bird’s eye view (triangle represents the participant’s nose) of the Part I tactor configurations; three tactors were placed on the torso (possible locations included the navel, spine, left and right side).

Vibrotactile stimuli were presented to four torso locations aligned with navel, spine, and the left and right sides of the torso at the level of the L4/L5 spinal segment. These specific four locations were selected for inclusion in this study because they are the most commonly used torso locations for balance-related applications [16,17,19]. Together, these three tactors and four locations formed four configurations as shown in Fig 1. The presentation order of configurations was randomized for each participant. All stimulus trials were 500 ms in duration. For each configuration, each type of vibrotactile stimuli was presented eight times [38]. The presentation order of these 32 stimulus trials (4 configurations × 8 stimulus trials) was randomized and then grouped into four blocks with eight stimulus trials per block. A randomized interval of 4–7 s was inserted between stimulus trials. Participants were asked to wear foam earplugs and earmuffs to eliminate the auditory cues generated by the vibration.

### Part II: Effects of stimuli locations and auditory cues generated by the vibration (ACV)

**Fig 2:**
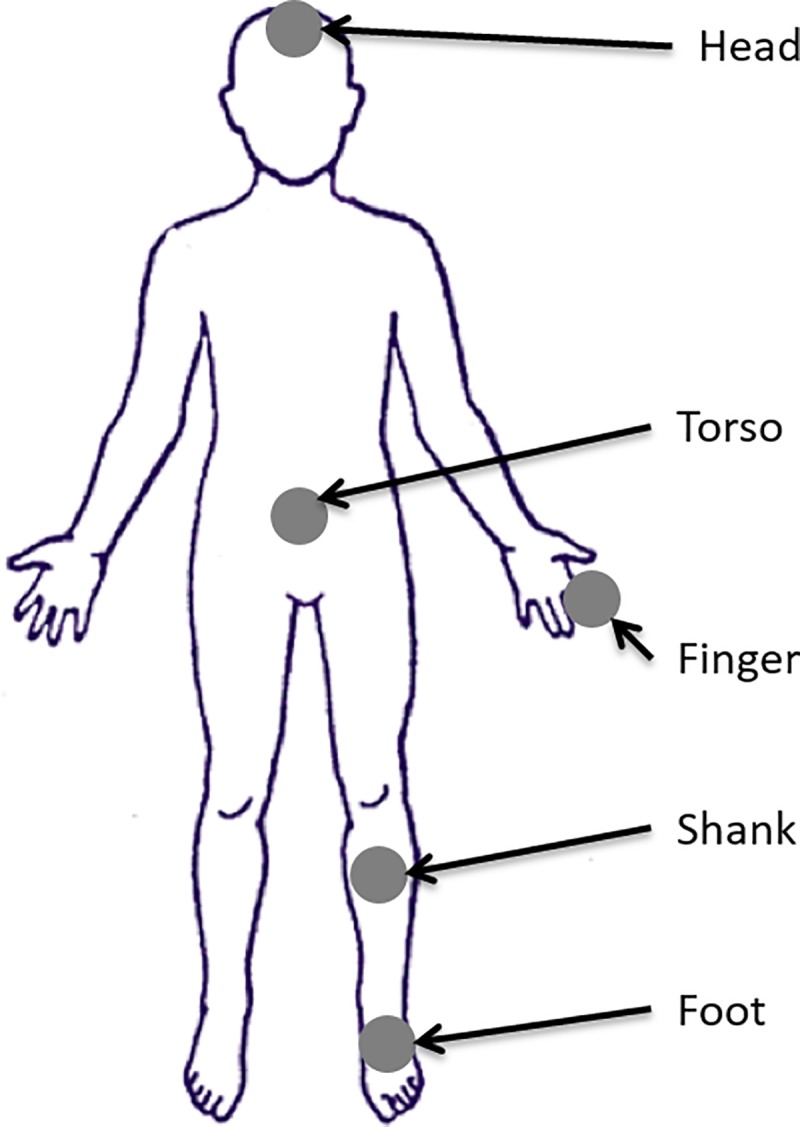
Experimental illustration for Part II: Five C-2 tactors were placed at five body locations.

The second part of the study tested the effects of tactor locations and the ACV on RTs. Vibrotactile stimuli were presented using C-2 tactors operating at 250 Hz at the following five locations on the body: center of the forehead (head), index fingertip on the dominant hand (finger), torso vertically aligned with the navel at L4/L5 spinal segment level (torso), anterior mid-shank on the dominant leg (shank), and dorsal base of metatarsus III on the dominant foot (foot) as shown in Fig 2. These five locations were chosen because they are commonly used in vibrotactile stimulation studies and applications [7,16,18,19,21–23]; 250 Hz was chosen because humans are most sensitive to vibrotactile stimuli applied with a frequency of 250 Hz [23,33]. Part II of the study was divided into two identical subparts: participants wore foam earplugs and earmuffs in the first subpart, but did not wear earplugs and earmuffs in the second subpart. For both subparts, all stimulus trials were 500 ms in duration and eight stimulus trials were performed per tactor, with a randomized interval of 4–7 s between stimulus trials. The presentation order of these 40 stimulus trials was randomized and then grouped into four blocks with ten stimulus trials per block.

### Part III: Effects of number of tactors in the same location

**Fig 3:**
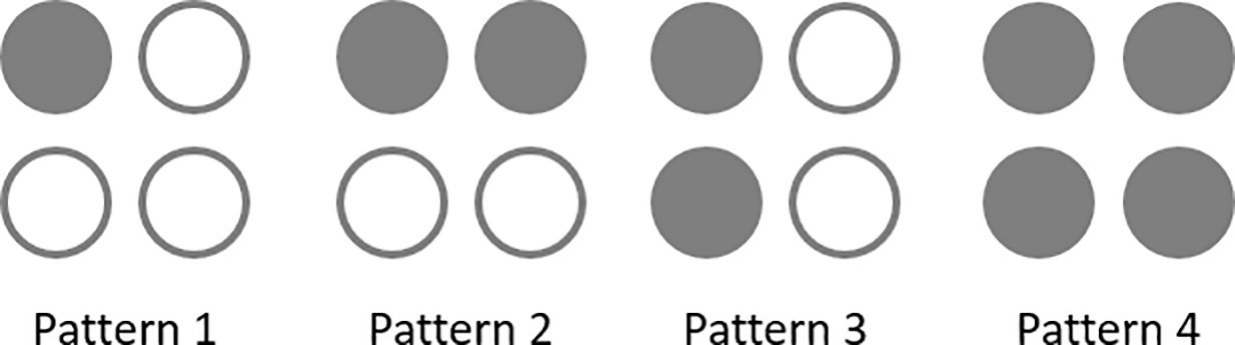
Experimental illustration for Part III: Four C-2 tactors grouped in a 2x2 array formed four patterns; the solid dots indicate the tactors that were activated in the corresponding patterns.

The third part of the study investigated the effects of the number of tactors closely grouped together in a given location on RTs. In this study, four C-2 tactors (operating at 250 Hz) were grouped in a 2x2 clustered array at the torso aligned with the navel at the L4/L5 spinal segment to form four vibration patterns as shown in Fig 3. The distance between the nearest edges of two adjacent tactors was 1 cm. In pattern 1, only one tactor in the upper left corner of the cluster was activated. In patterns 2 and 3, two tactors in the horizontal direction and vertical direction of the cluster were simultaneously activated, respectively. In pattern 4, all tactors within the cluster were activated simultaneously. A stimulus trial for this part of the study was defined as the presentation of a given pattern for 500 ms. The presentation order of these 20 stimulus trials (4 patterns × 5 stimulus trials) was randomized and then grouped into two blocks with 10 stimulus trials per block. Randomized intervals of 4–7 seconds separated each stimulus trial. In this part of the study, participants were asked to wear foam earplugs and earmuffs.

### Part IV: Effects of secondary task

The fourth part of the study explored the effects of simultaneously performing a secondary task on participants’ RTs to the vibrotactile stimuli. The tactor placement locations for this part of the study coincided with the tactor placement locations from Part II (i.e., five tactors were placed at the five locations shown in Fig 2); however, participants did not wear earplugs or earmuffs. During Part IV of the study, participants were asked to perform a secondary cognitive task, used to divide their attention and increase their cognitive load [39], while simultaneously performing the RT task. Specifically, participants were asked to continuously count backward by 3s from an initial number provided by a study team member (randomly assigned, ranging from 90 to 99) [40]. Verbal responses were audio-recorded. If participants counted down to 0 within any given period, they were asked to begin counting backwards from the initial number provided to them. Participants’ baseline performance on the cognitive task was initially assessed during a single 60-s period (matching the length of subsequent blocks) without the simultaneous performance of the RT task. After this initial assessment, 40 stimulus trials were randomized and then grouped into four blocks with ten trials per block. Participants were asked to perform the RT task while simultaneously performing the cognitive task throughout all stimulus trials.

### Data analysis

RTs faster than 100 ms were considered to be erroneous and were discarded [23,26,41–43]. The maximum RT was a function of the time interval between stimuli (e.g., 4–7 s) and was considered as a lack of response. The lack of a response prior to the subsequent stimulation was considered a missed response and was not included in the analysis. All data were logarithmically transformed to achieve a normal distribution prior to the analysis. Repeated measures of RTs for a given condition for each participant were averaged to obtain a single representative RT.

The differences in average RTs per condition between YA and OA were analyzed as two-tailed t-tests. To analyze the effects of different vibration factors on RTs, a linear mixed effect model (LMM) was used. The benefit of using a LMM was that it accounted for the correlation between multiple observations of the same subject [44]. A different LMM was used for each experimental part. Fixed factors included the type of vibrotactile stimuli (Part I), tactor location and presence of auditory cues generated by the vibration (ACV) (Part II); stimulation pattern (Part III), and location of tactors and presence of secondary task (Part IV). When only one factor was included in the model, it was considered a fixed effect factor. If two factors were included, these factors were combined as one factor which is considered as a fixed effect factor. The differences among participants were always considered as random effects for all analyses. The analysis was performed using R (r-project.org, package ‘lmerTest’ with t-tests using Satterthwaite’s method) and 95% confidence intervals (95% CI) were reported. The significance was defined by p-values less than 0.05. For multiple level pair-wise comparisons, the Bonferroni correction was applied.

The performance of the secondary cognitive task was evaluated by calculating the number of verbal responses per minute. The correctness of counting was not evaluated in this study. The significance was analyzed using the LMM by replacing ‘Reaction Times’ with ‘Number of Verbal Responses’.

## Results

Only one data point was faster than 100 ms; the participant mentioned that he might have accidentally pushed the thumb trigger during that trial. The slowest RT was found during the dual-task condition and was less than 2 s. All other RTs were less than 1 s for all parts of the study. For YA, 0.3%, 0% and 0% of RTs were considered as missing data points for Part I, Part II, and Part III, respectively. For OA, the missing data points increased to 2.3%, 0.5%, and 0.5% for Part I, Part II, and Part III, respectively; most of the missing RTs occurred when the participants were presented with stimuli at the spinal location during Part I. There were several possible reasons for missing points including: the participants were not able to sense the vibration, the participants did not push the trigger hard enough to trigger a response, and/or the stimulation system did not work properly. Only OA participated in Part IV. The percentage of missing data points increased to 8.9% for Part IV, where the stimulation at the foot location contributed to more than half (56%) of the total missing data points. An increase in missing data points was expected due to the dual-task paradigm. Among all OA participants, two out of the nine participants were more likely to miss stimuli than the other participants.

The results of tested factors were reported for each experimental part. In the text that follows, the RT values reported in the parentheses represent the differences between the stated comparisons; only significant differences are reported. In the case of a single comparison, a single RT value and its p-value are reported. In the case of multiple comparisons, a range of RT values representing the minimum and maximum values, and the least significant p-value among these comparisons are reported.

Across all experimental parts, RTs for YA were significantly faster than OA to the same type of vibrotactile stimuli (range: 31–122 ms, *p* < 0.01). It was also noted that that the variance of RTs within the OA population was significantly higher than within the YA population for the same vibrotactile stimuli (*p* < 0.001).

### Effects of frequency and types of tactors (Part I)

**Fig 4:**
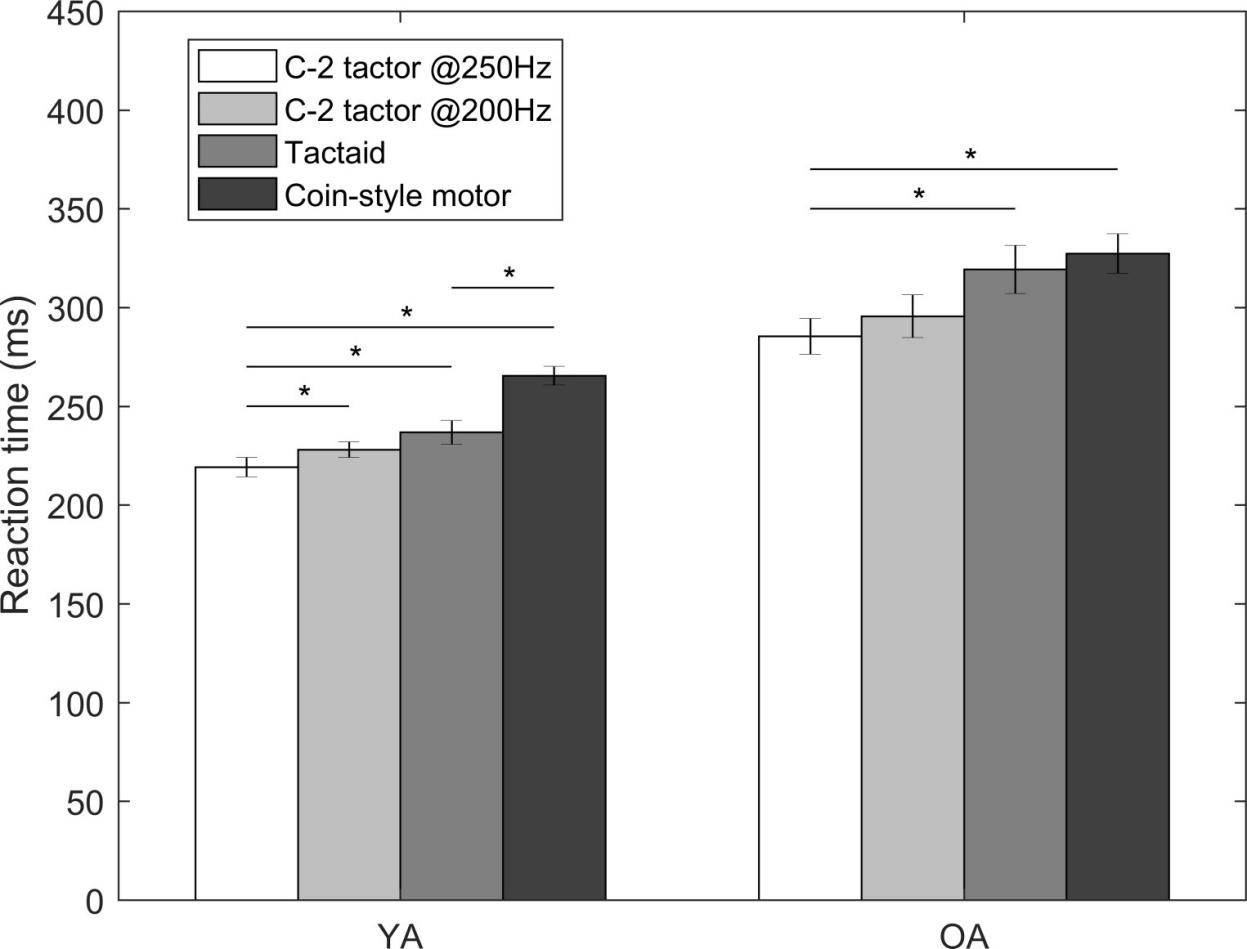
RTs to four different types of vibrotactile stimuli at torso locations; error bars present standard errors of means across participants; asterisk symbols (*) present statistical significance.

The mean RTs to the four types of vibrotactile stimuli are shown in Fig 4. Comparisons were made between the C-2 tactor operated at 250Hz versus 200Hz, and among the C-2 tactor operated at 250 Hz, the Tactaid and coin-style motors. RTs to the C-2 tactor stimulus operated at 250 Hz were significantly faster than RTs to the C-2 tactor stimulus operated at 200 Hz for YA (9 ms, 95% CI = [0.02, 0.07], *p* < 0.001), but no significant differences were observed between these two operating frequencies for OA. For both YA and OA, RTs to the C-2 tactor stimulus operated at 250 Hz were significantly faster than RTs to the stimuli provided by the Tactaid tactors (17 ms, 95% CI = [0.05, 0.10], *p* < 0.001 for YA; 34 ms, 95% CI = [0.11, 0.17], *p* <0.001 for OA) and coin-style motors (46 ms, 95% CI = [0.18, 0.22], *p* < 0.001 for YA; 42 ms, 95% CI = [0.11, 0.18], *p* <0.001 for OA). RTs to the stimuli provided by Tactaid tactors were significantly faster than RTs to the stimuli provided by coin-style motors for YA (29 ms, 95% CI = [0.10, 0.15], *p* <0.001), but no significant differences were observed between these two stimuli for OA.

### Effects of tactor locations and auditory cues generated by the vibration (ACV) (Part II)

**Fig 5:**
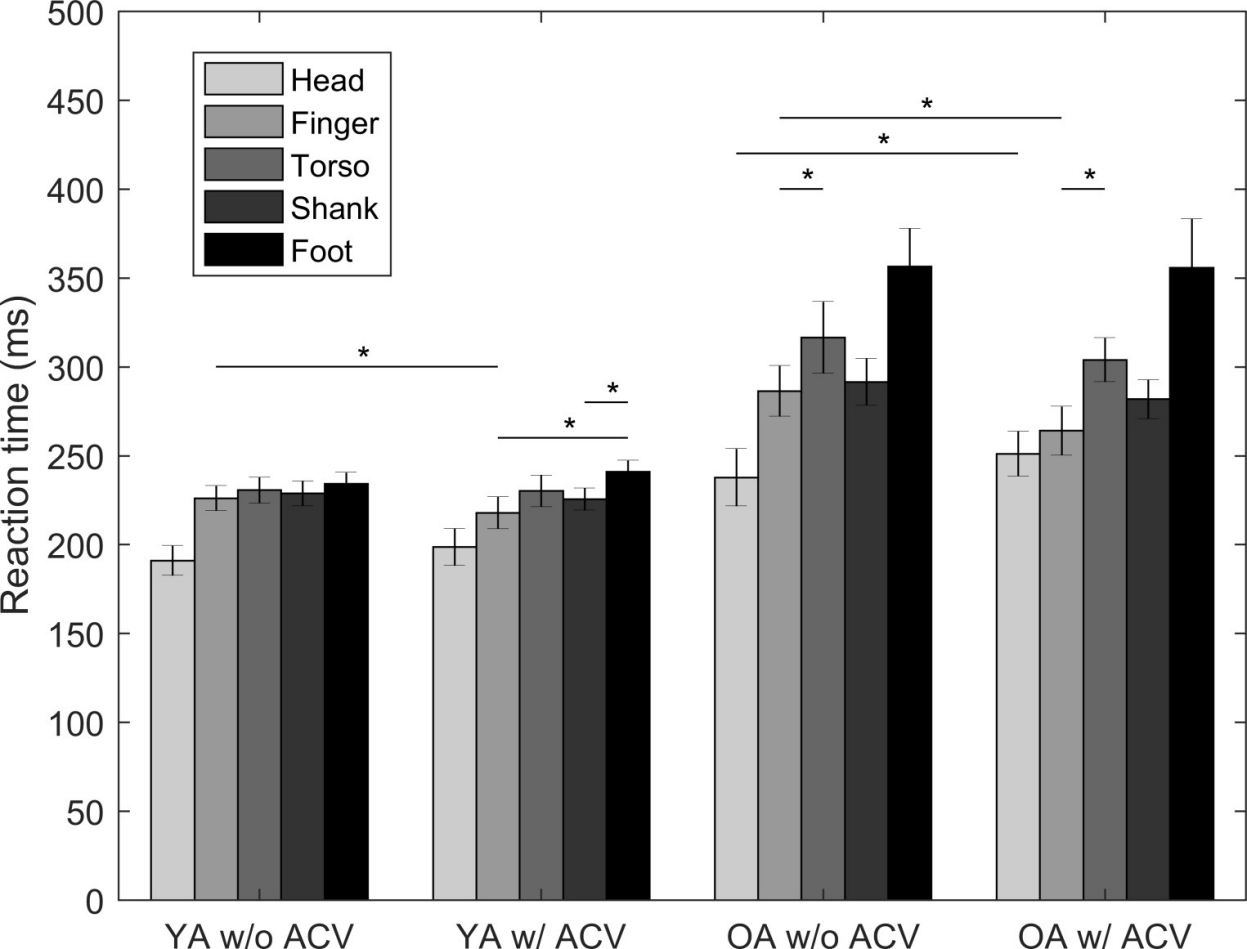
RTs to vibrotactile stimuli at five different body locations for both YA and OA with and without ACV; error bars present standard errors of the means across participants. Asterisk symbols (*) indicate a subset of the comparisons that were statistically significant; although significant, the following statistically significant comparisons are not indicated with asterisks on the figure: 1) RTs at the head location were significantly faster than all other locations for both YA and OA, except for the comparison between RTs at head and finger locations for OA, and 2) RTs at the foot location were significantly slower than all other locations for OA.

The mean RTs to the vibrotactile stimuli at the five body locations are shown in Fig 5.

Among YA, regardless of ACV, RTs were significantly faster at the head location than the other four locations (range: 35–43 ms, *p* < 0.001 without ACV; range: 19–43 ms, *p* < 0.001 with ACV). Without ACV, there were no significant differences among these four locations. With ACV, RTs were significantly slower at the foot location compared to the finger (23 ms, 95% CI = [0.07, 0.15], *p* < 0.001) and the shank (15 ms, 95% CI = [0.03, 0.11], *p* = 0.001) locations. Compared with RTs without ACV, RTs with ACV were significantly faster at the finger location (8 ms, 95% CI = [0.01, 0.09], *p* = 0.02); no significant differences were observed for the other four locations.

Among OA, regardless of ACV, RTs at the foot location were the slowest (range: 40–119 ms, *p* < 0.001 without ACV; 48–99 ms, *p* < 0.001 with ACV), and RTs at the torso location were significantly slower than RTs at the finger location (30 ms, 95% CI = [0.04, 0.16], *p* = 0.001 without ACV; 37 ms, 95% CI = [0.09, 0.20], *p* < 0.001 with ACV). Without ACV, RTs at the head location were the fastest (range: 49–119 ms, *p* < 0.001). With ACV, RTs at the head location were the fastest (range: 31–99 ms, *p* < 0.001) with the exception of the finger location. Compared with RTs without ACV, RTs with ACV were 1) significantly slower at the head location (13 ms, 95% CI = [0.002, 0.12], *p* = 0.04); 2) significantly faster at the finger location (22 ms, 95% CI = [0.02, 0.13], *p* = 0.01); 3) not significantly different among the other three locations.

### Effects of number of tactors in the same location (Part III)

**Fig 6:**
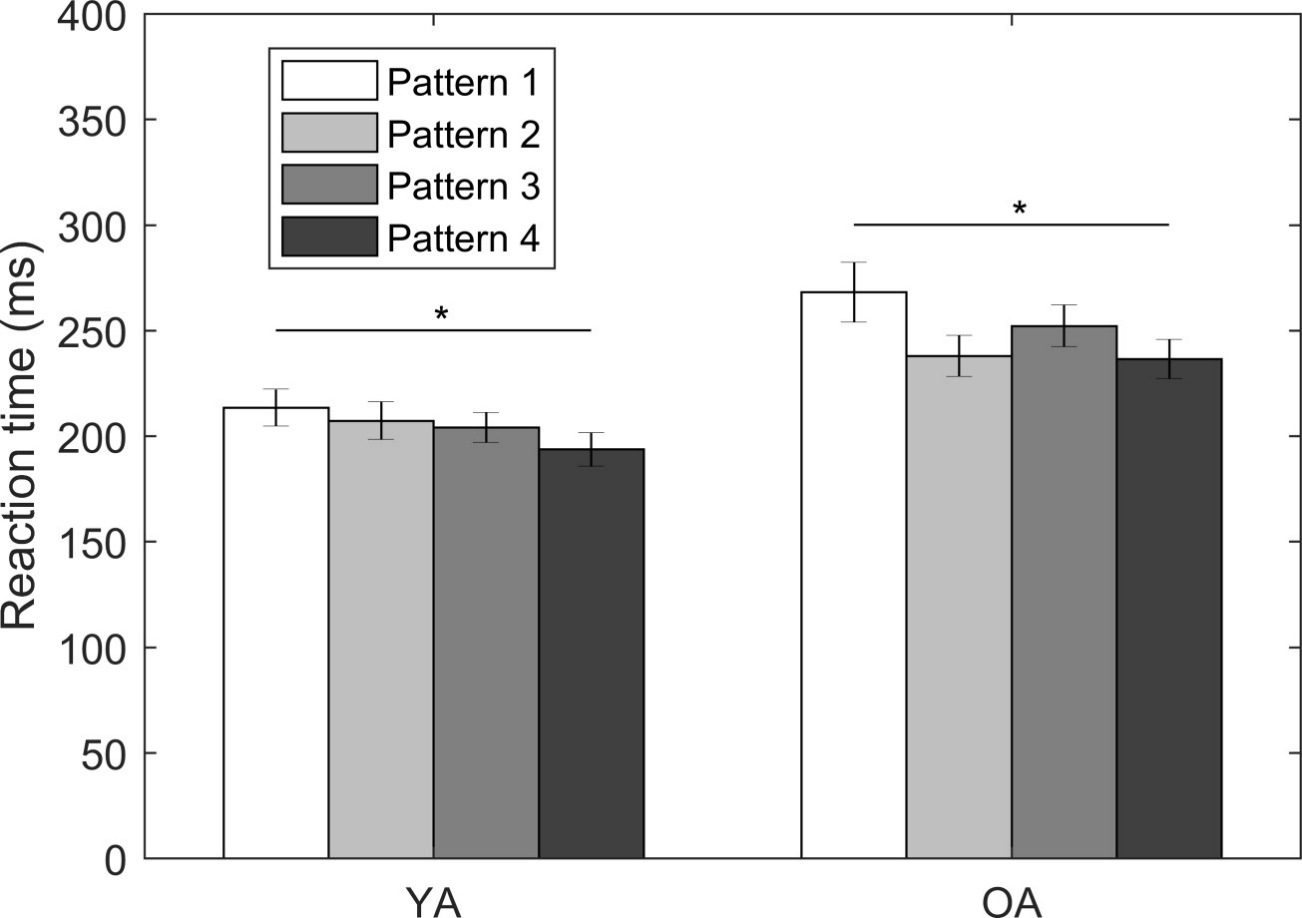
RTs to the four stimulation patterns for both YA and OA; error bars present standard errors of means across participants; asterisk symbols (*) present statistical significance.

RTs for the pattern-based vibrotactile stimuli presented at the navel location are shown in Fig 6. For both YA and OA, RTs to pattern 4 were significantly faster compared to pattern 1 (20 ms, 95% CI = [0.05, 0.15], *p* < 0.001 for YA; 32 ms, 95% CI = [0.04, 0.19], *p* = 0.004 for OA); however, there were no significant differences among the other three patterns.

### Effects of the secondary task (Part IV)

**Fig 7:**
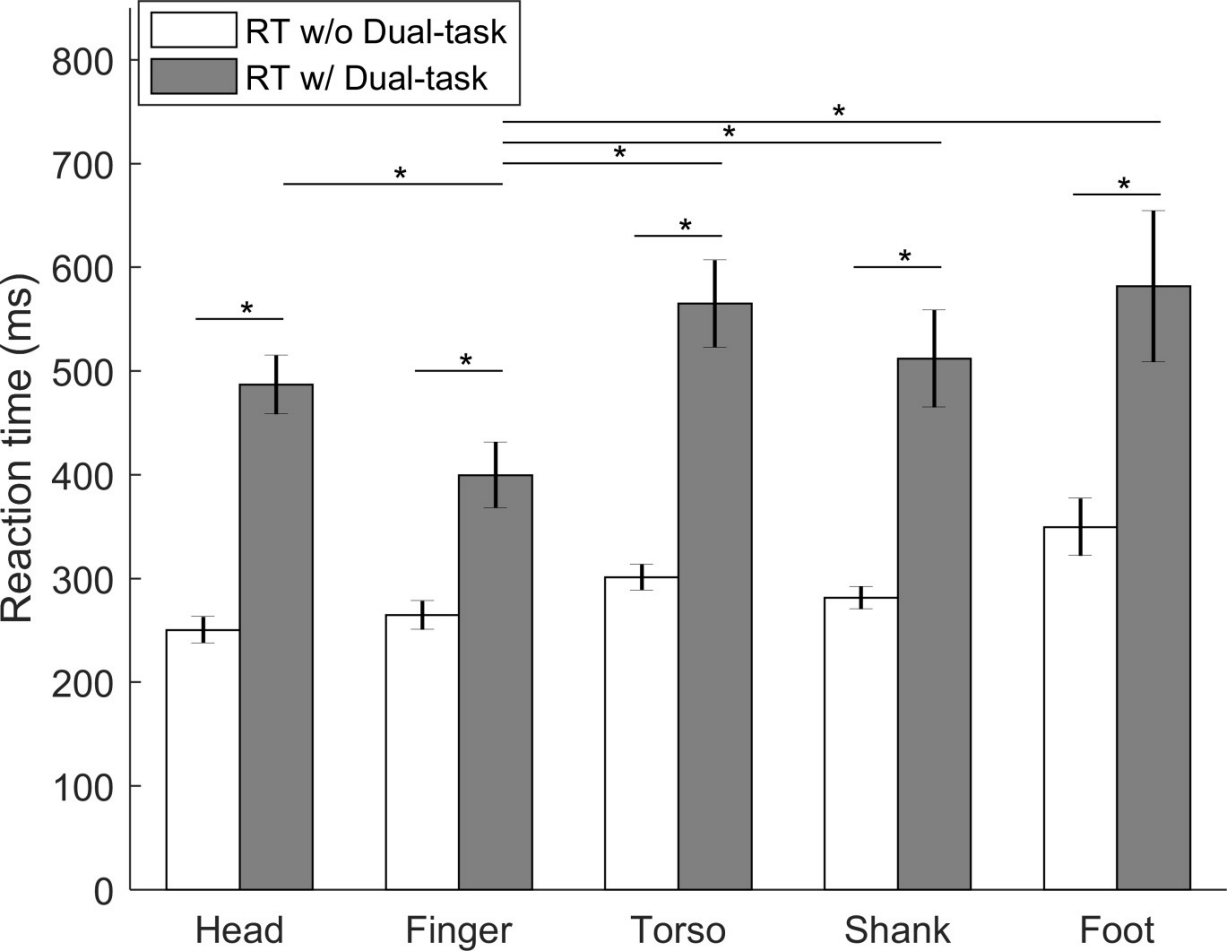
RTs to the vibrotactile stimuli at five different body locations with or without the secondary task; error bars present standard errors of means across participants; asterisk symbols (*) present statistical significance.

The effects of the secondary task on RTs to vibrotactile stimuli and the performance of the secondary task were examined. RTs for the nine participants who participated in Part IV are shown in Fig 7. With the secondary task, the RTs at the finger were significantly faster than the RTs at the other four locations (range: 87–182 ms, *p* < 0.001). RTs with and without the secondary task were compared for each location for the six participants who completed the experiments on both days. At all locations, RTs to vibrotactile stimuli significantly increased when the secondary task was performed (range: 135–264 ms, *p* < 0.001).

All nine participants had slower counting speeds when performing the two tasks simultaneously; on average, the number of verbal responses per minute significantly decreased from 39.7±2.5 to 34.1±1.8 (*p* < 0.01).

### Effects of number of possible stimulus locations

**Fig 8:**
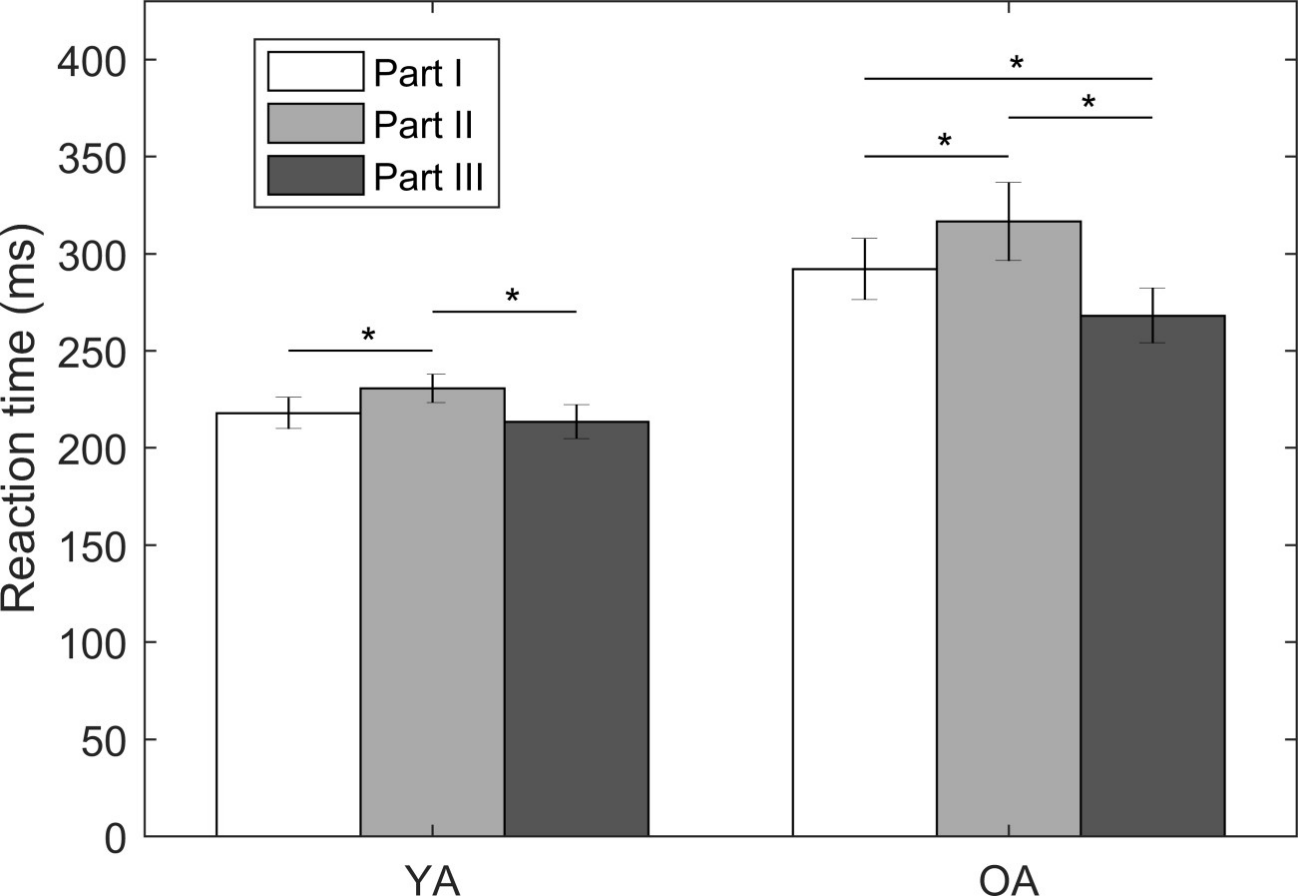
RTs for the common tactor located at the navel for three experimental parts; Part I: Three tactors at four locations around the torso; Part II: Five tactors at five locations across the body; Part III: Four tactors in a cluster at the navel. Error bars present standard errors of means across participants; asterisk symbols (*) present statistical significance.

By design, there was one common C-2 tactor placed at the navel location for Parts I-III (Part I: three tactors at four locations around the torso; Part II: five tactors at five locations across the body; Part III: four tactors in a cluster only at the navel location) to facilitate comparisons of RTs at the same location when the number of potential stimuli locations varied. The results are shown in Fig 8. Among both YA and OA, for the same tactor type (single C-2 tactor operated at 250 Hz) and location (navel location), RTs in Part II were significantly slower than RTs in Parts I (13 ms, 95% CI = [0.02, 0.10], *p* = 0.006 for YA; 24 ms, 95% CI = [0.02, 0.13], *p* = 0.01 for OA) and Part III (17 ms, 95% CI = [0.03, 0.12], *p* = 0.002 for YA; 48 ms, 95% CI = [0.10, 0.23], *p* < 0.001 for OA). For OA, the RTs in Part I were significantly slower than those in Part III (24 ms, 95% CI = [0.02, 0.15], *p* = 0.01).

### Summary of results

Overall, vibrotactile stimuli factors affected RTs by less than 10 ms to more than 250 ms. Table 2 summarizes the various factors’ effects (only includes statistically significant differences for comparisons among various factors) based on RTs for both YA and OA.

**Table 2:** Summary of various factors’ effects on RTs for YA and OA (only significant effects shown).

Factors	Comparisons	Absolute Difference in RTs	
		Young adults	Older adults
Stimulus frequency	250* vs 200 Hz	9 ms	N/S**
Auditory cues generated (ACV) by vibration (with ACV vs without ACV)	@ head location (without ACV*)	N/S**	22 ms
	@ finger location (with ACV*)	8 ms	13 ms
Number of tactors in the same location	One vs four*	20 ms	31 ms
Number of tactor locations	One* vs five	17 ms	48 ms
Type of tactors	C-2* vs Tactaid	18 ms	34 ms
	C-2* vs Coin-style motor	46 ms	42 ms
Stimulus location with ACV	Head* vs finger	19 ms	N/S**
	Head* vs torso	31 ms	51 ms
	Head* vs shank	27 ms	31 ms
	Head* vs foot	42 ms	99 ms
Stimulus location with secondary task	Finger* vs head	N/A^#^	87 ms
	Finger* vs torso		165 ms
	Finger* vs shank		112 ms
	Finger* vs foot		182 ms
Secondary cognitive task (with task vs without task*)	@ finger location	N/A^#^	135 ms
	@ head, shank, foot locations		233 ms
	@ torso location		264 ms

* faster condition

** not significant

^#^ secondary cognitive task was only tested on older adults

## Discussion

In this study, the effects of various factors, including vibration frequency, type of tactors, tactor location, auditory cues generated by the vibration (ACV), number of tactors in the same location and secondary task on RTs to vibrotactile stimuli were investigated for both healthy YA and OA. The differences among RTs between YA and OA were also studied.

### Young and older adults

On average, RTs for OA were 60 ms slower than the RTs for YA, which is in agreement with prior RT findings to audio-visual stimuli as a function of age [30]. This observed difference may result from age-related reduced neuron conduction rates [30,45], decreased cognitive and motor functions [46,47], and/or deteriorated sensory function [48]. However, in our study, RT differences between YA and OA were as large as 100 ms depending on the experimental condition. In general, differences in RTs between YA and OA increased when YA had large RTs. For example, YA reacted in ~200 ms to stimuli provided at the head location, while OA reacted in ~250 ms to stimuli at the same location (~50 ms difference); YA reacted in ~230 ms to stimuli at the foot location, while OA reacted in ~330 ms to stimuli at the same location (~100 ms difference).

### Stimulus frequency and number of tactors in the same location

RTs to changes in stimulus frequency and the number of tactors in the same location were predictable; the fastest RTs were observed when stimuli were operated at 250 Hz compared with 200 Hz and RTs decreased as the number of tactors in the same location increased. Pacinian Corpuscles have an optimal sensitivity at approximately 250 Hz [2,49–51], and sensitivity has been shown to increase as contact area increases for relatively small areas (< 5 cm^2^) [50]. Overall, these two factors have a relatively lower impact on RTs (i.e., 10–30 ms) compared with other factors investigated in this study.

### Type of tactors

RTs to the C-2 tactor were approximately 45 ms faster than the coin-style motor. However, the C-2 tactor is substantially more expensive (i.e., $250) than a coin-style tactor (i.e., $5). Thus, there is a trade-off between RT and cost.

### Stimulus location

Harrar and Harris [26] found that RTs to vibrotactile stimuli were linearly related to the distance from the forehead by 45 ms/m for YA. In this study, it was found that as the distance from the head to the tactor location increased, RTs generally increased except for RTs to stimuli applied at the torso. For both YA and OA, although the distance from the forehead to the torso was shorter than the distance from the forehead to the finger and the shank, RTs at the torso were slower than RTs at both the finger and shank. This counter-relationship of distance is likely due to greater skin sensitivity to vibration at the finger and shank than the torso [52]. In the study by Harrar and Harris [26], the tactors were placed on the forehead, lip, neck, finger and foot locations, which are generally more sensitive than the torso [52]. Thus, the RTs could be linearly related to the distance from the forehead for stimulus locations with comparable sensitivities.

By comparing RTs at the head, finger, shank and foot, a linear relationship with slopes of 26 ms/m for YA and 67 ms/m for OA was found, which is faster than the conduction velocities from Harrar and Harris [26]. However, converting our findings for conduction velocity from ms/m to m/s yielded 38 m/s for YA and 15 m/s for OA, which is closer to the conduction velocities measured using physiological techniques [53]. Finally, the slope (ms/m) for the OA was more than twice as steep as the slope for the YA. This implies that the effects of body location on RTs increases with age and that stimulus locations far from the forehead should be taken into careful consideration for certain applications, especially for OA.

### Auditory cues generated by the vibration (ACV)

The effects of ACV on RTs were different for different locations. RTs at the finger were significantly faster for both populations when ACV were provided, which is consistent with previous findings by Diederich and Colonius [54] who found that RTs to combinations of auditory and tactile stimuli were faster than RTs to each type of stimulus alone. However, the presence of ACV did not influence RTs at the torso, shank, or foot for either population. This may be caused by the contact medium (e.g., abdominal skin) and the distance between the sound source and ear. Interestingly, at the head location, wearing earplugs and ear muffs did not affect the RTs for YA, but reduced the RTs for OA. One potential reason for these findings is the occlusion effect, which assumes that bone conduction increases sensitivity to low frequency sounds when the ear canal is blocked [55,56]. Thus, when participants were wearing earplugs and ear muffs, the ACV was possibly magnified rather than blocked. Environmental noise might have interfered with the ACV when participants removed the earplugs and ear muffs.

### Secondary task

When a secondary task was performed, RTs to vibrotactile stimuli significantly increased by at least 130 ms compared to no secondary task. Slower RTs were coupled with poorer performance of the cognitive task. These findings agree with previous studies [20,57]. Mohebbi et al. [57] showed large increases in RTs (148 ms) to vibrotactile collision warnings when participants talked on a cell phone. Lin et al. also found a significant increase in RTs to a cognitive task when the participants were asked to perform balance exercises with vibrotactile cues [20].

During the presence of the secondary task, a linear relationship was not observed between RTs and the distance from the forehead; while performing the secondary task, RTs at the finger location were the fastest (there were no significant differences among RTs at the head, torso, shank, and foot locations). The RTs at the head and finger locations may be explained by the stimulus-response compatibility effects, which refer to how compatible the locations of stimuli are with the location of response [58–60]. According to Kornblum et al., the high compatibility sets should yield faster responses than the low compatibility sets [58]. In this study, the sets involved stimuli at the head and finger location, and responses at the thumb location with a trigger-push. The finger-thumb response set was more compatible than head-thumb stimuli-response sets which resulted in faster RTs at the finger. These results agree with those by Ho et al. where they found a set-level compatibility between stimulus locations of wrist and foot, and response locations of hand and foot [61]. However, this compatibility effect was not observed during the single task experiments (experimental Part II). One possible explanation is that the compatibility effect does not outweigh the effect of distance from the forehead, which was also found in the study by Ho et al. With the increased cognitive load by the addition of secondary task, the compatibility effect may have been magnified.

The presence of the secondary cognitive task also negatively affected the number of trials completed with appropriate RTs. Two prevalent attention division theories are the capacity-sharing theory (attentional capacity is limited and performing two tasks will cause deterioration of one or both tasks) and the bottleneck theory (one task will be delayed until the other task is completed) [39,62,63]. Aligned with the bottleneck theory, participants may have been processing the secondary cognitive task when the 50-ms vibration occurred and were not able to perceive and react to the vibration.

### Number of possible stimulus locations

RTs to a common location (navel) increased when there were more potential stimulus locations (five locations from head to foot in Part II, four torso locations in Part I and one navel location in Part III). Hick’s law applied to the present study predicts that RTs should increase as the number of stimulus locations increase. Donders’s subtraction method segments the reaction to vibrotactile stimuli into four stages: stimulus detection, stimulus discrimination, response selection and motion execution [42]. For recognition and choice RT experiments, it was shown that stimulus discrimination and response selection have larger effects on RTs compared to stimulus detection and motor execution [28,42,64]. In this study, participants were asked to push the trigger regardless of the stimuli location. We posit that the difference in RTs at the navel location is due to stimulus detection rather than stimulus discrimination, response selection, or motion execution. Participants’ attentional capacities may have been divided across multiple potential stimulus locations during Parts I and II, as compared to Part III and the stimulus detection process may have subsequently been delayed. The effect of age was also notable in this scenario; RTs at the navel for OA differed by ~50 ms between Part II (five stimulus locations) and Part III (single stimulus location), which were approximately three times larger than the differences observed for the YA (~15 ms).

### Applications

The results of this study should be considered and applied to the design of vibrotactile displays. Factors such as stimulus frequency, tactor type, number of tactors in the same location, stimulus location or age could play an important role in applications that require relatively high-frequency motions (i.e., less than 500 ms) including surgical applications [23], real-time gait training, golf training [24], or fall prevention [22]. For example, when changes were made in the experimental configurations used for investigating the effects of these factors on RTs in this study, RTs varied by as much as 50 ms, which is approximately 10% of the required time to complete the high-frequency motions. Furthermore, a longer RT should be expected if a vibrotactile display involves multiple stimulus locations. Thus, the number of stimulus locations should also be carefully considered.

For other applications where motions are generally more than 500 ms in duration, factors that affect RTs by less than 50 ms may not be as important, but factors such as stimulus location (i.e., head vs foot) or age should be considered because this study has shown that they can affect RTs by at least 100 ms. In applications where secondary cognitive tasks are likely to be performed such as in driving or flying [4,5,25], a significant increase in RTs should be expected as the results of this study demonstrated increased RTs of at least 130 ms when a cognitive task was performed.

This study also demonstrated that the selection of stimulus locations should be intentionally chosen to take into consideration delays in RTs as a function of the distance from the forehead, skin sensitivity, and stimulus-response compatibility. For example, in this study, faster RTs to stimuli were observed at the head location compared with the finger location when the participants were asked to perform a simple motor task (i.e., push a trigger using the thumb). However, when the secondary cognitive task was performed, faster RTs were observed at the finger location compared with the head location, potentially due to the higher compatibility between the stimuli-response set. In vibrotactile-based balance exercise training, the two most commonly used display locations to date have been the head [15,21] and the torso [16,65,66]. In this context, trainees typically use vibrotactile cues to prompt postural corrections of their center of mass (i.e., response location), which is approximately located at the level of the torso-based tactors used in this study. For a single motor task, like the task used in this study, RTs for the head location were faster than RTs the torso location. For this scenario, one might predict that the response-stimuli compatibility will yield RTs as fast if not faster than stimuli applied to the forehead despite the use of an inferior anatomical body segment.

Lastly, even though RTs to visual and auditory cues might elicit faster responses and potentially provide richer informative than vibrotactile cues [11], vibrotactile cues have distinct advantages within the fast-growing field of wearable technologies. Firstly, vibrotactile display have the potential to cause less interference with daily activities. For example, visual and auditory displays may not be suitable for use during scenarios involving simultaneous viewing or listening tasks. [7]. Secondly, vibrotactile displays may provide more flexibility for specific types of applications, e.g., for balance rehabilitation, vibrotactile displays can be used during eyes-closed and head movement exercise configurations [67]. Multi-modal displays may be appropriate in certain scenarios to address disadvantages of individual display modalities, however multi-modal displays may require more complicated and expense instrumentation [68].

### Limitations

There were several limitations of this study. Although contact pressure was roughly controlled between the skin and tactors using a shim, it likely varied somewhat within and across participants, which may have influenced the skin sensitivity and the perceived vibration intensity [69]. This limitation may have affected some of the findings in this study, including the effects of the stimulation frequency. The delay caused by the signal transmission was not measured given that the primary objective of the study was to elucidate relative differences in RTs to different vibrotactile display design factors; however, the lack of these data limits the interpretation of observed differences in RTs. In part III, the C-2 tactors used in this study may have been manufactured with different polarities thereby potentially affecting the results since RTs to parallel and anti-parallel vibrations might be different. Factors such as arousal [70,71], fatigue [71], prior experience, and physical parameters including height and weight were not controlled. The environmental noise level in the laboratory setting was not measured, which may have affected the results in Part I. This study did not investigate the effects of increasing increments of age on RTs, nor the effects of a secondary task on RTs for YA. Additionally, only three commonly used tactors were included in the comparison and only the C-2 tactor was used throughout all parts in this study. Finally, only simple responses to vibrotactile stimuli were studied as opposed to complex vibration patterns and alternative modes of response.
